# The complexity of the *Fragaria* x *ananassa* (octoploid) transcriptome by single-molecule long-read sequencing

**DOI:** 10.1038/s41438-019-0126-6

**Published:** 2019-04-06

**Authors:** Huazhao Yuan, Hongmei Yu, Tao Huang, Xinjie Shen, Jin Xia, Fuhua Pang, Jing Wang, Mizhen Zhao

**Affiliations:** 1 Institute of Pomology, Jiangsu Academy of Agricultural Sciences/Jiangsu Key Laboratory for Horticultural Crop Genetic Improvement, 210014 Nanjing, China; 2 Biomarker Technologies Corporation, 101300 Beijing, China; 3 Key Laboratory of Biology and Genetic Improvement of Oil Crops, Ministry of Agriculture of People’s Republic of China, Oil Crops Research Institute, Chinese Academy of Agricultural Sciences, 430062 Wuhan, People’s Republic of China

**Keywords:** Plant development, Sequence annotation

## Abstract

Strawberry (*Fragaria* x *ananassa*) is an allopolyploid species with diverse and complex transcripts. The regulatory mechanisms of fruit development and maturation have been extensively studied; however, little is known about the signaling mechanisms that direct this process in octoploid strawberry (*Fragaria* x *ananassa*). Here, we used long-read sequencing (LRS) technology and RNA-seq analysis to investigate the diversity and complexity of the polyploid transcriptome and differentially expressed transcripts along four successive fruit developmental stages of cultivated strawberry. We obtained a reference transcriptome with 119,897 unique full-length isoforms, including 2017 new isoforms and 2510 long noncoding RNAs. Based on the genome of the plausible progenitor (*Fragaria vesca*), 20,229 alternative splicing (AS) events were identified. Using this transcriptome, we found 17,485 differentially expressed transcripts during strawberry fruit development, including 527 transcription factors (TFs) belonging to 41 families. The expression profiles of all members of the auxin, ABA pathway, and anthocyanin biosynthesis gene families were also examined, and many of them were highly expressed at the ripe fruit stage, strongly indicating that the role of those genes is in the regulation of fruit ripening. We produce a high-quality reference transcriptome for octoploid strawberry, including much of the full-length transcript diversity, to help understand the regulatory mechanisms of fruit development and maturation of polyploid species, particularly via elucidation of the biochemical pathways involved in auxin, ABA, and anthocyanin biosynthesis.

## Crop genetics: Gene expression during strawberry ripening

Researchers in China have identified the genes involved in strawberry ripening. Mizhen Zhao led a team at the Jiangsu Academy of Agricultural Sciences which used long-read sequencing to investigate gene expression during six stages of strawberry fruit development, from small green fruit through whitening to large red fruit. They identified thousands of genes which are activated or repressed during fruit development, including 527 transcription factors – genes which regulate the activity of other genes. The analysis also revealed that genes related to the plant hormone auxin were downregulated in developing fruits, while those related to abscisic acid were upregulated, clarifying the role of the two hormones during strawberry ripening. This work not only provides a high-quality reference transcriptome for future strawberry work but also highlights genes related to ripening for further study.

## Introduction

Cultivated strawberry (*Fragaria x ananassa*) is an economically important soft fruit species that is grown commercially around the world^1^. Strawberry fruit undergo a series of dramatic changes during fruit ripening, including fruit size, texture, aroma, flavor, and color changes^2^. According to the changes in the respiration rate of fruits after harvest, fruits can be divided into two categories, climacteric and nonclimacteric^3,4^. Strawberry is a typical nonclimacteric fruit, as the respiratory burst and rise in ethylene production are absent during ripening^5^. It is believed that auxin and ABA, rather than ethylene, play essential roles in the regulation of strawberry fruit ripening^6–8^.

Cultivated strawberry (*Fragaria* x *ananassa*) originated in the 1700s from a natural hybridization between two octoploid strawberries, *F. virginiana* and *F. chiloensis*^9^. There is no high-quality reference genome or annotation for *Fragaria* x *ananassa* due to heterozygosity and complexity of the polyploid genome, and only a virtual reference draft genome is available^10^. Polyploidy and alternative splicing can greatly increase transcript diversity in eukaryotes^11^. This diversity may include alteration, reduction, or loss of function to influence post-transcription expression^12,13^. All of these influences contribute to the diversity and complexity of a polyploid transcriptome. However, little information is available on this for most genes in polyploid strawberry. Because of the ability to generate large amounts of sequence data in a relatively short amount of time, next-generation sequencing (NGS) technology has been widely used to quantify gene expression in recent years. Some studies have used RNA-seq technologies to analyze *Fragaria* x *ananassa* fruit development, but due to the lack of a high-quality transcriptome reference, the matching ratios are not ideal when mapping the transcriptomes^14,15^. NGS does have several significant limitations, especially short read lengths and amplification biases, which are particularly challenging for polyploid genomes^16^. Long-read sequencing (LRS) technology overcomes these difficulties by generating long reads (e.g., those exceeding 10 kb) without the need for further assembly, making it possible to accurately reconstruct full-length splice variants^17,18^. This technique has been applied to discover further information on novel genes and alternatively spliced isoforms in several species, including sorghum, maize, strawberry (*Fragaria vesca*), moso bamboo, cotton, and coffee bean ^19–23^.

This study used PacBio Iso-Seq analysis to establish a high-quality reference transcriptome for cultivated strawberry (*Fragaria* x *ananassa*), which provides not only valuable resources for exploring the complex polyploid system but also a dissection of many metabolic and signaling pathways, particularly hormone metabolism and anthocyanin biosynthesis pathways.

## Results

### Overview of transcripts from single-molecule long-read sequencing

We combined total RNA in equal amounts from five tissues, including mature leaf, flower, dwarf stem, root, and receptacle tissues, from six different stages (small green, middle green, large green, white, turning, and red) to acquire accurate full-length cultivated strawberry (*Fragaria* x *ananassa*) transcripts. After 51,060 short sequences (<300 bp) were removed, four single molecular real-time (SMRT) cells generated 1,095,914 reads of inserts, and 57% (627,654) of them were full-length nonchimeric reads with poly(A) tail signals, 5′ adaptor sequences and 3′ adapter sequences (Table 1). The length of FLNC reads ranged from 300 to 7248 bp, with an average length of 1989 bp. The length distribution of all FLNC reads is presented in Supplementary Information 1: Figure S1.

**Table 1:** Statistics of PacBio single-molecule long-read sequencing data

Library	1–2K	2–3K	3–6K	All
Reads of insert	291,926	493,489	361,559	1,146,974
Number of five prime reads	203,147	285,207	269,716	758,070
Number of three prime reads	211,807	296,372	274,323	782,502
Number of poly-A reads	212,615	289,476	273,135	775,226
Number of filtered short reads	13,324	25,795	11,941	51,060
Number of non-full-length reads	104,095	241,544	112,825	458,464
Number of full-length reads	174,507	226,150	236,793	637,450
Number of full-length nonchimeric reads	172,707	222,883	232,064	627,654
Average full-length nonchimeric read length	1521	2183	2153	1989
Full-Length percentage (FL%)	59.78%	45.83%	65.49%	55.58%

The PacBio platform has a higher sequencing error rate than the Illumina platform; thus, it is necessary to perform error correction, which includes self-correction by iterative clustering of circular-consensus reads and correction with high-quality second-generation sequencing (SGS) short reads. Therefore, we generated 96,317,763 clean reads using the Illumina platform to correct the single-molecule long-reads. The 627,654 full-length nonchimeric reads were further clustered into a total of 297,020 consensus transcripts. Combining the non-full-length reads and Illumina short-reads, the consensus transcripts were corrected by SMRT Analysis and proofread. The iterative cluster procedure and error correction resulted in many redundant transcripts. After removing redundant transcripts using CD-HIT 4.6.1 software and a manual filtering method, the number of error-corrected FLNC reads (named SMLR) was reduced to 119,897, each of which represents a unique full-length transcript.

### Identification and characterization of isoforms and alternative splicing

**Fig. 1:**
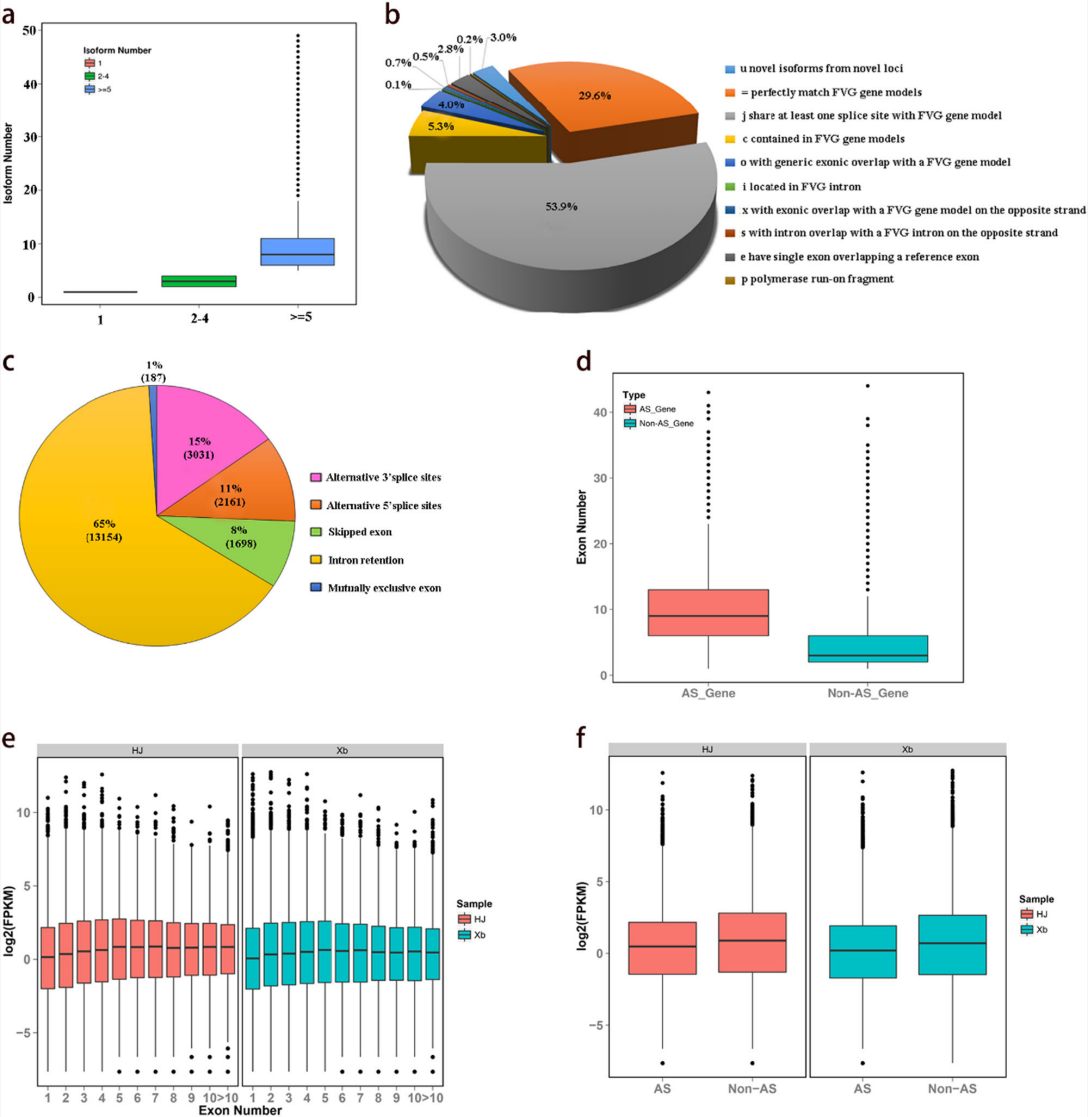
Identification and characterization of isoforms and alternative splicing. **a** Boxplot showing the percentage of *Fragaria vesca* loci mapped with different numbers of SMLR isoforms. **b** Comparison of SMLR isoforms with *Fragaria vesca* Genome v4.0.a1 by Cuffcompare. **c** Pie chart showing the number and percentage of AS events obtained from the SMRT. **d** Boxplot showing the exon numbers of AS and non-AS genes in SMRT. **e**. Boxplot showing the expression levels of SMRT isoforms with a different number of exons in the ripe strawberry fruit of two varieties (HJ and Xb). **f**. Boxplot showing the expression levels of AS and non-AS genes in the ripe strawberry fruit of two varieties (HJ and Xb)

The diploid wild strawberry (*Fragaria vesca*) is one of the plausible progenitors of *Fragaria* x *ananassa*, and its genome is small (~240 Mb) and well annotated^24,25^, which facilitates the analysis of transcripts in cultivated strawberry. We compared SMLR isoforms against the *Fragaria vesca* genome v4.0.a1(FVG), and 102,340 (85.4%) isoforms of SMLR were aligned to the FVG by GMAP. Summarizing the numbers of isoforms, 97,789 isoforms were successfully assigned to 16,126 *Fragaria vesca* genes, covering 56% of *Fragaria vesca* loci with an average of 6.06 isoforms. We detected 7628 genes with only a single isoform. In 5845 genes, two to four isoforms were assigned. For 7204 genes, 5 or more splice isoforms were mapped (Fig. 1a). However, a previously published *Fragaria vesca* PacBio Iso-Seq with 32,583 transcripts could be aligned to 13,960 *Fragaria vesca* genes, with an average of 2.33 isoforms per gene^17^. We made a direct comparison of SMLR and *Fragaria vesca* PacBio Iso-Seq and found more isoforms in *Fragaria* x *ananassa* than in *Fragaria vesca* for the same genes (Supplementary Information 1: Figure S2). These results showed that the transcript data of octoploid strawberry (*Fragaria* x *ananassa*) were more complex than those of diploid wild strawberry *Fragaria vesca* at the whole-genome level.

The aligned isoforms were then classified by Cuffcompare into 10 groups as follows (Fig. 1b): (a) 3051 (2.98%) novel isoforms were from novel loci. Using the BLASTX program (e-value ≤ e^−10^), we compared these novel isoforms to the proteins in related species, such as *Malus* x *domestica*, *Pyrus communis*, *Prunus persica*, and *Prunus avium*; 2375 (77.84%) isoforms were found to be homologous with at least one genome, and 2175 (71.29%) isoforms could be considered homologous in all four species; the remaining 676 isoforms (22.16%) had no homologues in any of the four species. (b) A total of 30,244 (29.55%) isoforms perfectly matched the FVG gene models. The transcripts in SMLR were longer than in the transcriptomes of the FVG gene models. The average length and N50 of these isoforms were almost twice as long as the FVG gene models (Supplementary Information 1: Figure S3). (c) A total of 55,139 (53.88%) novel isoforms shared at least one splice site with an FVG gene model but differed at other splice sites. Among these, 50,375 (91.36%) isoforms were homologous to annotated proteins in other Rosaceae. (d) The next group comprises 5419 (5.30%) isoforms contained in FVG gene models. Among these, 5205 (96.05%) were also shorter than the annotated proteins in other Rosaceae, suggesting that they may potentially be incomplete transcripts produced by sequencing. (e) This group comprises 4126 (4.03%) isoforms with generic exonic overlap with an FVG gene model. (f) A total of 107 (0.10%) isoforms were located in FVG introns. (g) A total of 703 (0.69%) isoforms had an exonic overlap with an FVG gene model on the opposite strand. (h) Four hundred seventy-four (0.46%) isoforms had an intron overlap with an FVG intron on the opposite strand. (i) Next, 2896 (2.83%) isoforms were potential premRNA fragments. (j) Finally, 181 (0.18%) isoforms were potential polymerase run-on fragments.

AS events have been investigated in *Fragaria vesca*^17^. However, the complexity of AS in allopolyploid strawberry (*Fragaria* x *ananassa*) remains largely unclear because of the high complexity of the polyploid transcripts. Using the Transcriptome Analysis Pipeline for Isoform Sequencing (TAPIS), 20,229 AS events were identified without transcriptome assembly, including 187 mutually exclusive exons, 13,154 intron retentions, 1696 skipped exons, 2161 alternative 5′ splice sites, and 3031 alternative 3′ splice sites (Fig. 1c). Consistent with reports in other plant species, intron retentions were the most frequent AS events^17^. In addition, there was a positive correlation between the number of exons and isoforms, and this correlation was more pronounced in AS genes, with a Pearson correlation coefficient of 0.59 (*P*-value = 0). We can see that the average number of exons in AS genes is larger than that in non-AS genes, but there was no significant difference between the two sets of data (*t*-test, *P*-value = 0.16) (Fig. 1d). Correlational analyses indicated that there was no significant difference between the isoform numbers and the expression levels (FPKM) of each gene in ripe strawberry fruit (Fig. 1e). The mean expression levels of AS genes were slightly lower than those of non-AS genes in strawberry (Fig. 1f).

### Long noncoding RNA identification

**Fig. 2:**
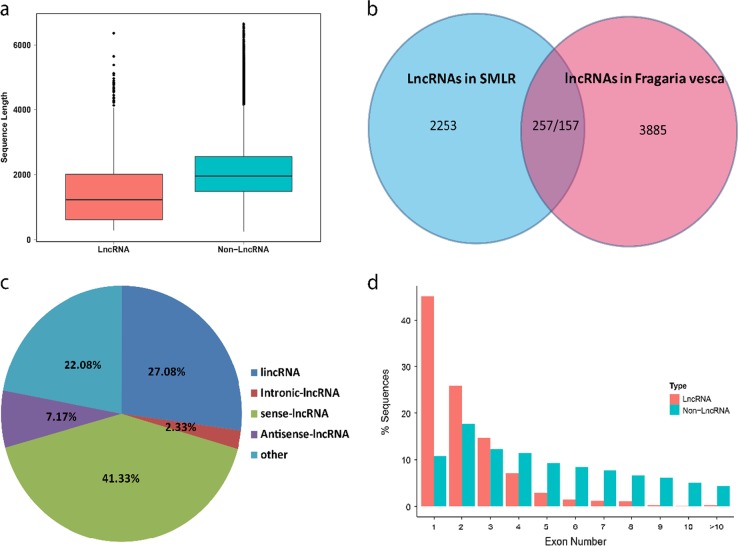
Long noncoding RNA from SMRT. **a** Boxplot showing the sequence length of lncRNA and nonlncRNA in SMLR. **b**. Venn diagram showing the number of common and unique lncRNAs detected in *Fragaria* x *ananassa* and *Fragaria vesca*. **c** Classification of 1,200 aligned lncRNAs in SMLR based on the location and transcription direction relative to adjacent protein-coding genes. **d** Distribution of exon number of lncRNAs and nonlncRNA in SMLR

Long noncoding RNAs (lncRNAs) have been shown to play critical regulatory roles in most eukaryotes. We built a combination of four pervasive coding potential assessment approaches (CPC, CNCI, CPAT, and Pfam) to identify lncRNAs from SMLR isoforms. A total of 2510 isoforms were predicted to be lncRNAs for further analysis, with no coding potential according to all four approaches. The lncRNA lengths varied from 285 to 6,364 bp, and the mean length was 1421.5 bp, which was much shorter than the length of nonlncRNAs in SMLR (*t*-test, *P*-value = 0.009) (Fig. 2a). In a previous study, a total of 1938 lncRNA loci were identified in the *Fragaria vesca* genome^26^. Consistent with this result, 1200 (47.81%) lncRNAs in SMLR were aligned to the *Fragaria vesca* genome, and the other 1310 lncRNAs may be specific to *Fragaria* x *ananassa*. Compared with previously discovered lncRNAs in Fragaria vesca, only 257 *Fragaria* x *ananassa* lncRNAs corresponded to 157 *Fragaria vesca* lncRNAs (Fig. 2b). The lncRNAs showed great variability in the two strawberry species, and polyploidy and alternative splicing did not increase the diversity or complexity of lncRNAs in strawberry. We then classified 1200 aligned lncRNAs into four groups based on their positions relative to the *Fragaria vesca* genome: 27.08% of the lncRNAs were generated from intergenic regions, 7.17% of the lncRNAs were generated from the antisense strand, 41.33% of the lncRNAs were generated from the sense strand, and 2.33% of the lncRNAs were generated from intronic regions (Fig. 2c). Interestingly, lncRNAs had fewer exons than nonlncRNAs; 542 (45.17%) of the lncRNAs had a single exon (Fig. 2d).

### Functional annotation

Functional annotation of isoforms in SMLR was conducted according to sequence similarities using seven databases (COG, GO, KEGG, KOG, Pfam, Swissprot, eggNOG, NR). As the data show in Table 2, a total of 115,370 (98.3%) isoforms were annotated after filtering out 2510 candidate lncRNAs, and the remaining 2017 isoforms had no hits in any of the above databases (Supplementary Information 2).

**Table 2:** Annotation results of isoforms in SMLR based on different databases

Anno database	Annotated_number	length ≥ 300 bp and <1000 bp	length ≥ 1000 bp
COG_annotation	45,394	2702	42,692
GO_annotation	68,316	6138	62,178
KEGG_annotation	48,458	3791	44,667
KOG_annotation	67,926	4499	63,427
Pfam_annotation	97,133	6624	90,509
Swissprot_annotation	83,417	6478	76,939
eggNOG_annotation	107,514	8775	98,739
nr_annotation	114,409	9859	104,550

We identified 114,409 (97.46%) and 83,417 (71.06%) isoforms that had at least one hit in NR and Swissprot, respectively. Of the species that harbored the best blast hits for SMLR isoforms in the NR database, the top ten species were *Fragaria vesca*, *Prunus mume*, *Prunus persica*, *Fragaria* x *ananassa*, *Malus domestica*, *Pyrus* x, *Fragaria chiloensis*, *Vitis vinifera*, *Medicago truncatula*, and *Populus trichocarpa*. Most hits found in *Fragaria vesca* (104,063, 90.69%) were likely identified because it is one of the progenitors of *Fragaria x ananassa* and is well annotated. There were only 1298 (1.13%) protein sequence hits found for *Fragaria x ananassa*, indicating that the NR database contains limited information on *Fragaria x ananassa* (Supplementary Information 1: Figure S4).

To remove redundancy, the functional classifications of SMLR were then performed at the gene level. After filtering out the candidate lncRNAs, 96,589 isoforms of SMLR were aligned to 16,009 *Fragaria vesca* genes and 1516 new gene loci by GMAP and gffcompare (v0.9.8). First, GO analysis was conducted using the Blast2GO program. A total of 9569 genes with 58,365 (59.68%) isoforms were successfully assigned to GO glossaries with 4041 functional terms. The genes were annotated in three categories: cellular component (CC, 5199 genes with 32,432 isoforms), molecular function (MF, 7642 genes with 48,143 isoforms), and biological process (BP, 7436 genes with 46,638 isoforms) (Supplementary Information 1: Figure S5). Within these functional groups, the most abundant functional groups in SMLR were as follows: cell part (4274 genes 44.67%), catalytic activity (5072 genes, 53.00%), and metabolic process (6481 genes, 67.73%).

To further define the pathways that the SMLR take part in, a BLAST analysis was performed against referenced canonical pathways in the KEGG database. We identified 6394 genes with 41,421 (42.36%) isoforms that annotated with 3084 KEGG identifiers (KOs) in the 120 KEGG pathways (Supplementary Information 1: Figure S6). Among these, the largest functional pathways were as follows: ribosome, with 235 KOs with 1044 isoforms (4.44 isoforms per gene) annotated, followed by biosynthesis of amino acids (175 KOs with 1470 isoforms, 8.4 isoforms per gene), plant hormone signal transduction (172 KOs with 1141 isoforms, 6.63 isoforms per gene), spliceosome (152 KOs with 1036 isoforms, 6.82 isoforms per gene), protein processing in endoplasmic reticulum (148 KOs with 1115 isoforms, 7.53 isoforms per gene), plant-pathogen interaction (128 KOs with 877 isoforms, 6.85 isoforms per gene), RNA transport (108 KOs with 850 isoforms, 7.87 isoforms per gene), endocytosis (101 KOs with 810 isoforms, 8.02 isoforms per gene), and carbon metabolism (99 KOs with 861 isoforms, 8.70 isoforms per gene).

### Temporal or spatial expression trends during strawberry (*Fragaria* x *ananassa*) fruit development

**Fig. 3:**
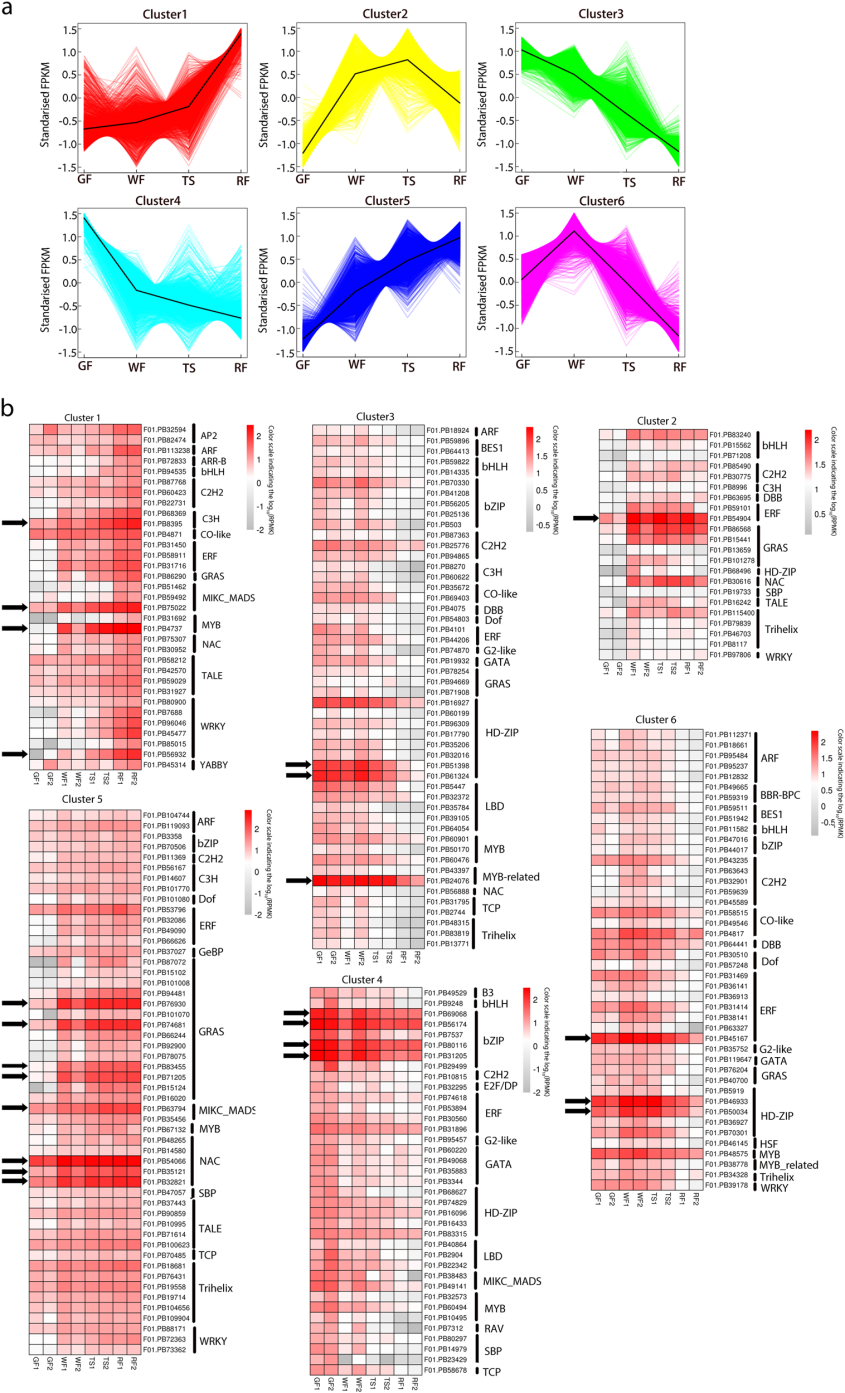
K-means clustering analysis of transcripts differentially expressed during strawberry fruit development. **a** Six K-means clusters of 13,755 isoforms showing differential expression during strawberry fruit development. **b** Heat map showing log_10_ (RPKM) for major TFs that were differentially expressed during strawberry fruit development and had RPKM values of 10 or higher at any of the four fruit development stages

To identify key genes involved in strawberry fruit development, we identified 15,514 (12.94% SMLR isoforms) differential expression isoforms in six combinations (GF-WF, GF-TS, GF-RF, WF-TS, WF-RF, TS-RF) based on the RNA-seq data (Supplementary Information 3). Among all isoforms differentially expressed during fruit development, most (12,945) isoforms were differentially expressed between GF and WF, and the smallest number of isoforms (210) were differentially expressed between WF and TS. Using the K-means clustering algorithm, 13,755 differential expression isoforms were grouped into six clusters (cluster1–6), representing 88.66% of all differential expression isoforms (Fig. 3a). To better understand the functions of isoforms that were differentially expressed in different stages of strawberry fruit development, enriched gene ontology (GO) enrichment analysis was performed (Supplementary Information 4). In cluster 1 and cluster 5, isoforms showed the lowest expression at the GF stage and gradual upregulation from the WF to the RF stage. Based on their GO terms, many isoforms in these two clusters were related to fatty acid, flavor, anthocyanin, and sugar biosynthetic processes. In contrast, cluster 3 and cluster 4 showed gradual downregulation from stage GF to RF, and many isoforms in these two clusters were related to the chlorophyll biosynthetic process, the photosystem and positive regulation of anthocyanin and carbohydrate metabolic processes. The fewest GO terms (530) were found for the transcripts in cluster 2, which showed high expression in the WF and TS stages. In contrast, isoforms in cluster 6 were highly expressed only in the WF stage.

Among the 13,755 genes that comprised the six superclusters, 527 were TFs belonging to 41 families (Supplementary Information 5). Due to the dominant expression of most duplicated genes in polyploid strawberry, we examined in more detail the major TFs with RPMK values of 10 or higher in any of the four fruit stages (Supplementary Information 5). These TFs showed distinct stage-specific expression patterns mirroring each of the six clusters from which they came (Fig. 3b). Most TFs belonged to cluster 3 (139) and cluster 4 (121), in which TFs had decreased expression from the GF stage to the RF stage and reached the lowest value in the RF stage; these included *bZIP44* (for BASIC LEUCINE ZIPPER 44) (F01. PB69068, F01. PB56174, F01. PB80116 and F01. PB31205), *HB5* (for HOMEOBOX PROTEIN 5) (F01. PB51398 and F01. PB61324), and *CPC* (for CAPRICE) (F01.PB24076). TFs belonging to cluster 1 (62) and cluster 5 (83) had increased expression from the GF stage to the RF stage, in which peak expression of the TFs was recovered in the RF stage. In particular, *ZFN3* (for ZINC-FINGER NUCLEASE 3) (F01. PB8395), four *SCL8* (for SCARECROW-LIKE 8) (F01. PB76930, F01. PB74681, F01. PB83455 and F01. PB71205), *SHP2* (for SHATTERPROOF 2) (F01. PB75022 and F01. PB63794), *PAP2* (for ANTHOCYANIN PIGMENT 2) (F01. PB4737), three *NAC2* (for NAC-REGULATED SEED MORPHOLOGY 2) (F01. PB54066, F01. PB35121 and F01. PB32821), and *WRKY28* (F01. PB56932) transcripts were mainly expressed in the red fruit (RF) stage and very rarely present in the early fruit development stage (GF and WF). This result suggests that TFs play an important role in strawberry fruit ripening, similar to their *Arabidopsis* homologues^27–29^. Cluster 2 (37) and cluster 6 (85) contained fewer TFs, which were most highly expressed in the WF and TS stages; for example, these included two ethylene responsive element binding factor *ERF3* genes (for AP2/ETHYLENE-RESPONSIVE FACTOR) (F01. PB54904 and F01. PB45167) and two *HB7* genes (F01. PB46933 and F01.PB50034).

### Auxin and ABA pathway gene dynamics during fruit development

After eliminating the isoforms with a premature stop by alternative splicing events, the SMLR isoforms mapped to those *F. vesca* genes were identified as strawberry (*Fragaria* x *ananassa*) auxin and ABA pathway transcripts. Phylogenetic analysis using *Fragaria* x *ananassa*, *F. vesca*, and *Arabidopsis* auxin and ABA pathway protein sequences showed that abundant homologous genes existed in octoploid strawberry (*Fragaria* x *ananassa*) (Supplementary Information 1: Figure S7 and S8).

**Fig. 4:**
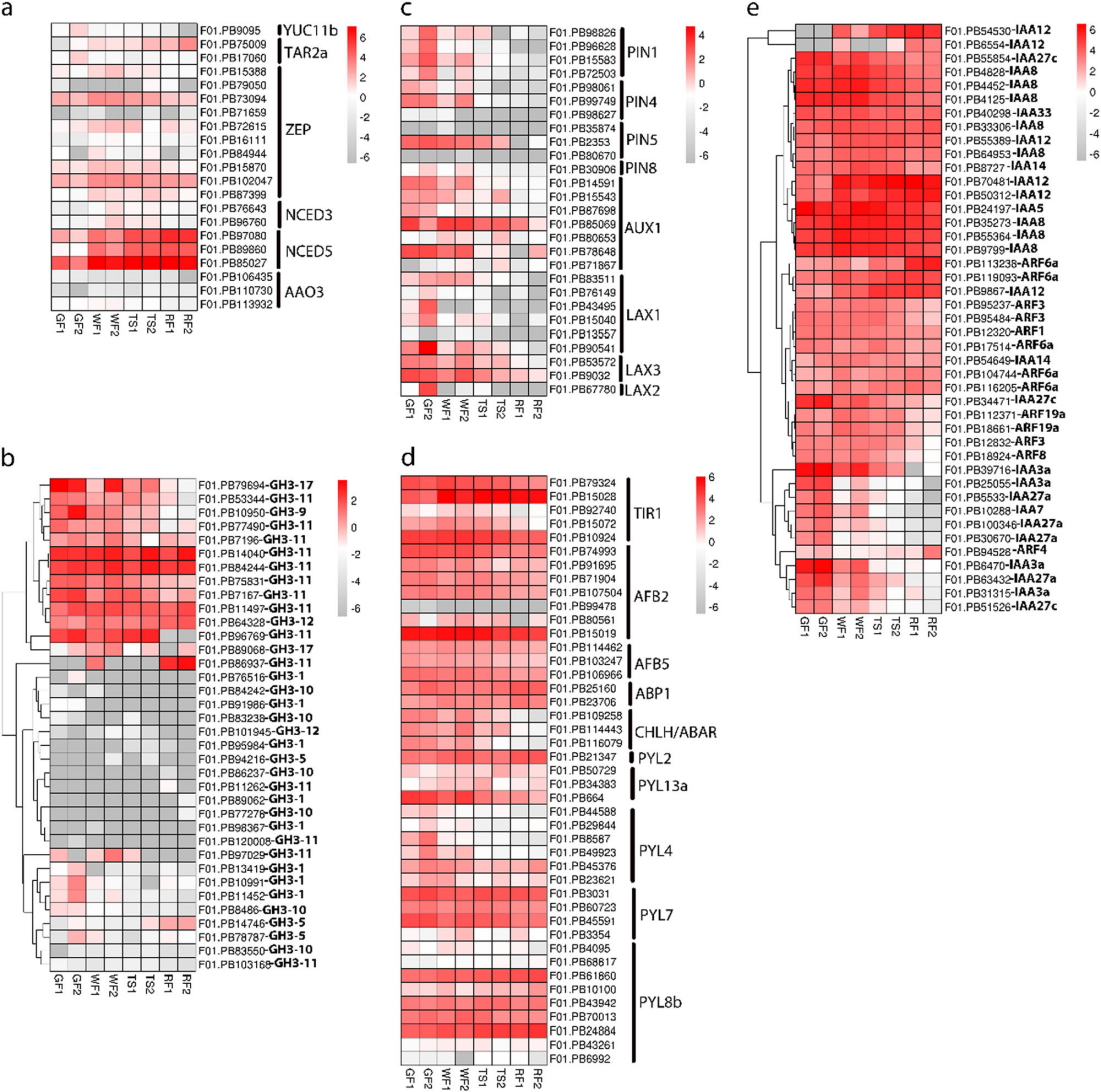
Heat map showing log_2_ (RPKM) values of expression for major auxin and ABA pathway-related genes during strawberry fruit development. **a**. Biosynthesis genes. **b**. GH3 genes. **c**. Auxin efflux and influx transporters. **d**. Auxin and ABA receptor genes. **e**. ARF and AUX/IAA genes with RPKM values of 10 or higher in any of the four fruit stages

The majority of auxin biosynthesis gene families (*YUC*, *TAA*, and *TAR*) were not detected in the corresponding transcripts in SMLR, or the corresponding transcripts were lowly expressed in the strawberry fruit (receptacle) during the whole growth and ripening process, which indicated that the receptacle was not the site of IAA biosynthesis^30,31^. In contrast, the corresponding transcripts of most ABA biosynthesis gene families (*ZEP*, *NCED*, and *AAO*) were contained in SMLR. In particular, *NCED5* (F01. PB85027) was highly expressed from the WF stage to the RF stage and may play an important role in the regulation of strawberry fruit ripening (Fig. 4a, Supplementary Information 6).

The auxin-responsive GH3 gene family can reduce free indole-3-acetic acid (IAA) by conjugating excess IAA to amino acids^32^. Because of low levels of auxin, all members of the *GH3* gene family were expressed at low or undetectable levels in strawberry fruit (receptacle) (Fig. 4b). Similarly, auxin efflux and influx transporters (*PIN*, *AUX/LAX*) had very low expression in strawberry fruit, and most of them declined during the ripening process (Fig. 4c).

The expression of auxin receptor genes (*TIR*, *AFB*, and *ABP*) showed downward regulation during the strawberry fruit ripening process, but the dependent relationships were not significant. For example, *AFB2* (F01. PB15019) exhibited relatively high expression in the GF stage and a gradual decline during the ripening process. An exception was *TIR1* (F01. PB15028), which had lower expression in the GF stage and four-fold higher expression in the TS and RF stages. The ABA receptor gene family (*PY*L) also had relatively low expression in strawberry fruit, but *PYL8b* (F01. PB24884) showed slightly higher expression in ripe strawberry fruit (Fig. 4d).

*ARFs* (122) and *AUX/IAAs* (73) are a large group of gene families related to auxin signaling that showed transcriptomic diversity and complexity in the octoploid strawberry. In particular, the protein-coding loci *gene06434-FvARF2* corresponded to 34 transcripts in SMLR (Supplementary Information 6). Overall, our data show that the AUX/IAAs were expressed at higher levels than ARFs in strawberry fruit (Fig. 4e). Because of gene expression redundancy due to polyploidy, we examined in more detail the major *ARFs* and *AUX/IAAs* with RPMK values of 10 or higher in any of the four fruit stages. Multiple members of the *AUX/IAAs* family, such as *IAA3a* (F01. PB6470 and F01. PB39716), *IAA8* (F01. PB55364, F01. PB9799, F01. PB35273), *IAA12* (F01. PB50312), and *IAA5* (F01. PB24197), were highly expressed in strawberry fruit. Biochemical and genetic studies in *Arabidopsis* have shown that Aux/IAA proteins dimerize with ARF activators to repress their activity^33,34^. In our work, the coexpression pattern of various strawberry *AUX/IAA* and *ARF* members indicated potential functional pairs, such as *IAA12* (F01. PB9867) with *ARF6a* (F01. PB119093) and *IAA14* (F01. PB54649) with *ARF6a* (F01.PB104744).

### Global analysis of anthocyanin biosynthesis genes

The most apparent ripening-related change is the color alteration in strawberry fruit. Anthocyanins are the major pigments responsible for the color of strawberry fruits. Due to transcriptome sequence diversity and alternative splicing of the polyploid system, the number of anthocyanin biosynthesis transcripts in *Fragaria* x *ananassa* is much larger than in *F. vesca*. From our transcriptome (SMLR), 83 anthocyanin biosynthesis transcripts from 15 families were identified, including two R2R3-MYB members (Supplementary Information 7).

**Fig. 5:**
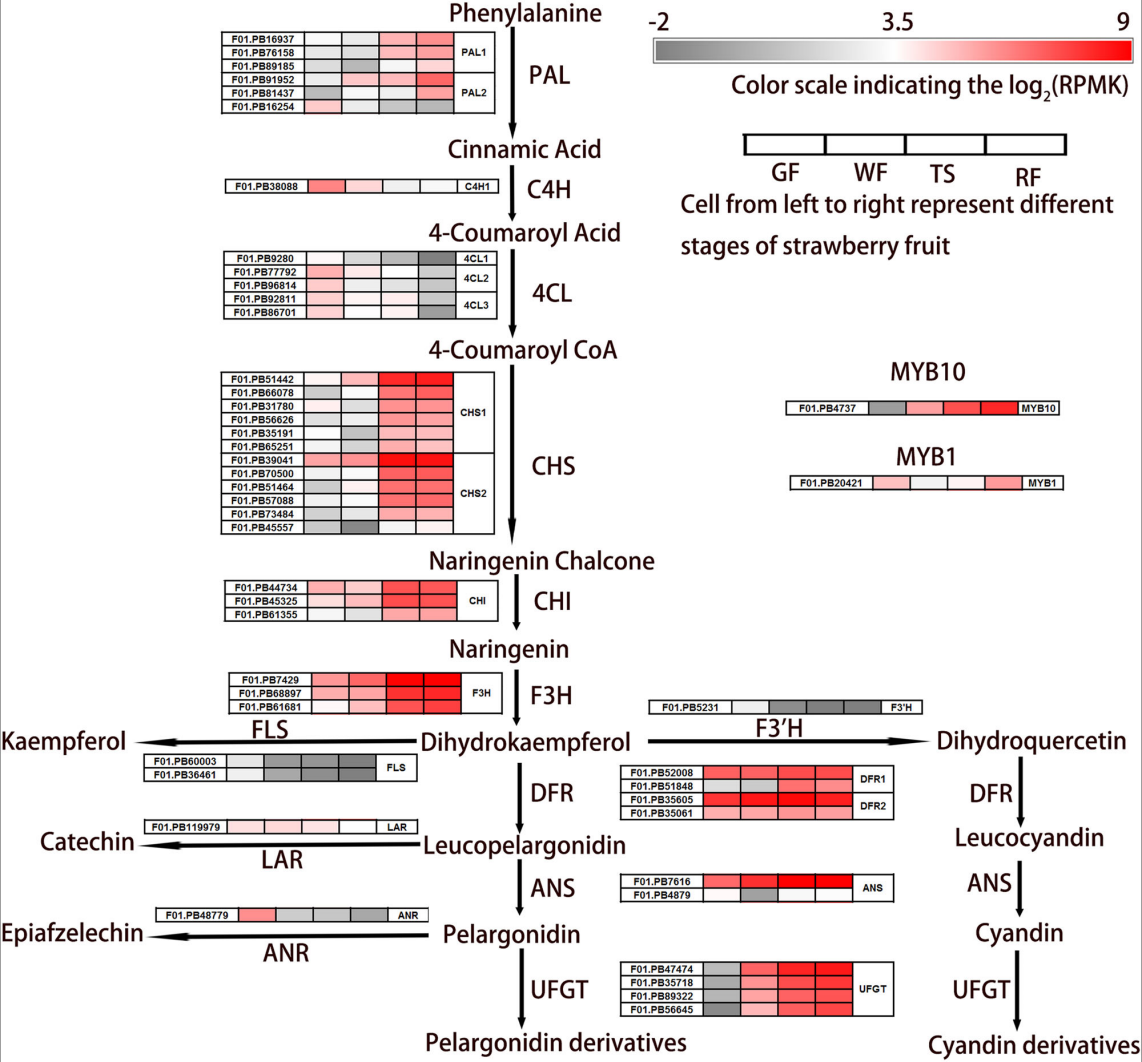
Schematic of anthocyanin biosynthesis pathways in strawberry fruit. Heat maps depicting log_2_ (RPKM) values for anthocyanin biosynthesis transcripts at the side of each step that had RPMK values of 10 or higher in any of the four fruit stages (GF, WF, TS, and RF). *PAL* Phe ammonia-lyase, *C4H* Cinnamate-4-hydroxylase, *4CL* 4-coumaroyl-CoA synthase, *CHS* Chalcone synthase, *CHI* Chalcone isomerase, *F3H* Flavanone 3-hydroxylase, *F3′H* Flavonoid 3′-hydroxylase, *ANS* Anthocyanidin synthase, *FLS* Flavonol synthase, *DFR* Dihydroflavonol 4-reductase, *UFGT* Anthocyanidin 3-O-glucosyltransferase, *LAR* Leucoanthocyanidin reductase; ANR: Anthocyanidin reductase

We examined in more detail anthocyanin biosynthesis transcripts with RPKM values of 10 or higher in the four strawberry fruit transcriptomes (Fig. 5). The majority of anthocyanin biosynthesis transcripts showed low or absent expression at early fruit developmental stages (GF and WF) and rapid increases at the anthocyanin accumulation stages (TS and RF); these included *PAL*, *CHS*, *CHI*, *F3H*, and *UFGT*. In contrast, *C4H* and *4CL*, which are involved in 4-coumaroyl CoA synthesis, showed gradual decreases in expression from high expression at the GF stage to low expression at the RF stage. Flavonols and flavan-3-ols are competitive synthetic branches for anthocyanin biosynthesis in the flavonoid pathway. *FLS*, *LAR*, and *ANR* are key enzyme genes in flavonols and flavan-3-ols, which showed a decreasing expression pattern during strawberry fruit ripening. *DFR* and *ANS*, two late-stage anthocyanin biosynthetic genes, showed very high expression levels during the entire fruit development period. *F3′H* catalyzes the first step in the cyanidin derivative biosynthesis branch and determines the cyanidin content^35^. *F3′H* was rarely expressed during the entire strawberry fruit development period. This result was consistent with the anthocyanin component of strawberry fruit, which mainly contains pelargonidin derivatives and a small number of cyanidin derivatives. The R2R3-MYB transcription factors play a vital role in the regulation of the flavonoid pathway. It was previously found that *FaMYB10* was specifically expressed in ripe fruit and was involved in regulating early and late-stage anthocyanin biosynthesis genes, while *MYB1* regulated anthocyanin biosynthesis in strawberry fruit as a transcriptional repressor of anthocyanin biosynthesis^28^. In our results, *MYB10* showed the same expression tendencies as the majority of anthocyanin biosynthesis transcripts, which had a gradual decrease of expression during strawberry fruit development and a high expression at the TS and RF stages, while *MYB1* showed relatively low expression and was mainly expressed at the GF and RF stages. In summary, anthocyanin biosynthesis transcripts were more highly expressed at the anthocyanin accumulation stages, particularly at the ripe fruit stage. The data strongly indicate that these genes regulate anthocyanin accumulation.

## Discussion

Over the past decade, NGS technology has become an integral part of genetic research and discovery. Many studies have generated a reference transcriptome in model eukaryotic species by NGS, but this has proven more challenging for complex polyploid genomes. In polyploid species, homologous genes often transcribe highly similar isoforms. There is currently no high-quality reference genome or annotation for octoploid strawberry (*Fragaria* x *ananassa*), which hinders utilization of these data for the accurate analysis of gene function. LRS technology can generate long reads without the need for further assembly, providing an excellent opportunity to accurately reconstruct full-length splice variants.

Our experimental design aimed to maximize transcript diversity and investigate the mechanisms of fruit ripening by broadly sampling five tissues, including mature leaf, flower, dwarf stem, root, and receptacle tissues from six different stages. LRS generated 1,095,914 reads of inserts, and 57% (627,654) of these were full-length nonchimeric reads, nearly double that of previous studies of its plausible progenitor’s (*Fragaria vesca*) transcriptome^17^. This study established a high-quality reference transcriptome for cultivated strawberry (*Fragaria* x *ananassa*) for the following reasons. First, the isoforms had an average length of 2100 bp in SMLR, which was much longer than in the RNA-Seq and EST data sets (RefTrans^36^, 1324 bp, is a transcriptome for *Fragaria* x *ananassa*) (Supplementary Information 1: Figure S9). Second, the SMLR showed a higher completeness level. In BUSCO alignment to 1440 conserved proteins, the SMLR isoforms had 90.3% (92.9%, including fragmented isoforms) BUSCO completeness (Supplementary Information 1: Figure S10). In the end, the trimmed reads of the RNA-seq datasets, including four successive developmental stages of strawberry (*Fragaria* x *ananassa*) fruit, had a perfect match with SMLR isoforms (Supplementary Information 1: Table S1). Although our SMLR isoforms only cover 56% of *Fragaria vesca* loci, *Fragaria vesca* genes on average mapped with 6.06 SMLR isoforms per gene, demonstrating that SMRT sequencing is very sensitive for uncovering new isoforms. The only downside is that the sample tissues and the number of SMRT cells might not have been enough to efficiently uncover low-abundance isoforms, but the sequencing depth was sufficient to explore as many full-length splice isoforms as possible considering the small strawberry genome size and in comparison with previous similar studies of SMRT transcriptomes.

Polyploidy and alternative splicing can greatly increase transcript diversity in eukaryotes^11^. In this study, the average gene mapped with 6.06 SMRT isoforms based on the *Fragaria vesca* genome, quadrupling the number in the *Fragaria vesca* annotation (v2.0.a2). For 7204 genes, 5 or more splice isoforms were mapped (Fig. 1a). However, few additional AS events were discovered in *Fragaria* x *ananassa* compared to *Fragaria vesca*, which may be due to the omission of AS events generated by homologous genes. LncRNAs are rapidly evolving and are often species specific^12^. Our results showed that only 1200 (47.81%) lncRNAs could be aligned to the *Fragaria vesca* genome (Fig. 2b).

Despite the regulatory mechanisms of fruit development and maturation having been studied extensively, the molecular mechanisms that direct this process in octoploid strawberry (*Fragaria* x *ananassa*) remain unclear. We found 17,485 differentially expressed transcripts during strawberry fruit development, and 88.66% of them grouped into six clusters (clusters 1–6) by the K-means clustering algorithm (Fig. 3a). Based on enriched gene ontology (GO) enrichment analysis, many differentially expressed transcripts belonged to fatty acid, flavor, anthocyanin, photosystem, and carbohydrate metabolic processes, which influence a series of dramatic changes in fruit size, texture, aroma, flavor, and color (Supplementary Information 4). Fruit ripening is a complex developmental process that includes fruit size, texture, aroma, flavor, and color changes, and hormones and transcription factors play important roles in this process^37^. In this study, 527 transcription factors (TFs) belonging to 41 families were differentially expressed during strawberry fruit development (Supplementary Information 5). Furthermore, the distinct red fruit stage-specific expression patterns of some TFs suggest that they may play important roles in fruit ripening, such as *ZFN3*, *SCL8*, *SHP2*, *PAP2*, *NAC2*, and *WRKY28* (Fig. 3b). It was previously found that *PAP2* was specifically expressed in ripe fruit and involved in the regulation of strawberry pigmentation biosynthesis^38^. Previous studies using DR5 reporters showed that auxin abounded in the seed and was absent from the pericarp, suggesting that seeds are the predominant source of auxin, rather than pericarp^39,40^. Our data also show low or even absent expression levels of auxin biosynthesis genes (*YUC*, *TAA*, and *TAR*) in the strawberry fruit (receptacle) during the entire growth and ripening process (Fig. 4a, Supplementary Information 6). Furthermore, auxin receptors (*TIR*, *AFB*, and *ABP*) and signaling components (*ARFs* and *AUX/IAAs*) showed strong expression in the receptacle (Fig. 4d, e), which indicates that auxin is likely transported to the receptacle and plays an important role in stimulating receptacle growth. By contrast, most ABA biosynthesis gene families (*ZEP*, *NCED*, and *AAO*) had higher expression levels in strawberry fruit (receptacle) when compared with the auxin biosynthesis genes, especially *NCED5* (F01. PB85027) (Fig. 4a). Downregulation of *FaNCED1* using the TRV-mediated VIGS technique could result in a significant decrease in ABA levels and uncolored fruits^7^. According to the above results, ABA may be synthesized in the receptacle and positively regulate strawberry ripening.

The anthocyanin biosynthesis pathway is well understood and is conserved among seed plants. A total of 83 anthocyanin biosynthesis transcripts from 15 families were identified from our transcriptome, which is much more than in *F. vesca* (Supplementary Information 7). The majority of anthocyanin biosynthesis transcripts, such as *PAL*, *CHS*, *CHI*, *F3H*, *DFR*, *ANS*, *UFGT*, and *MYB10*, showed very high expression in ripening strawberry fruit. Transcripts involved in competitive branches for anthocyanin biosynthesis, such as *FLS*, *LAR*, and *ANR*, showed a decreasing expression during strawberry fruit ripening (Fig. 5). As is generally known, *F3′H* catalyzes the first step in the cyanidin derivative biosynthesis branch and determines the varieties of anthocyanins. RNAi-mediated suppression or overexpression of *F3′H* can clearly change cyanidin-derived anthocyanins in plants^41,42^. Our results showed that the lack of cyanidin derivatives could be due to the low expression of *F3′H* in strawberry fruit (Fig. 5).

In summary, our work establishes a high-quality polyploid strawberry reference transcriptome using LRS technology. The transcriptome contains diverse and complex transcript isoforms that were generated from polyploidy and alternative splicing. Using this reference, comprehensive and genome-wide studies of biochemical pathways were conducted in polyploid strawberry, including auxin, ABA, and anthocyanin biosynthesis pathways. Some transcripts were found to exhibit distinct stage-specific expression patterns, which are potential gene resources to help understand the regulatory mechanisms of fruit development and ripening.

## Materials and Methods

### RNA sample preparation

Strawberry plants were grown in the plastic greenhouse at Jiangsu Academy of Agricultural Sciences. The fruit receptacles were frozen in liquid nitrogen immediately and stored at −80 °C after being cut into small pieces. The other tissues were directly frozen in liquid nitrogen and stored at −80 °C until further use.

Total RNA was extracted using Tiangen RNA preparation kits (Tiangen Biotech, Beijing, China) following the provided protocol. RNA integrity was assessed using a Nanodrop ND-1000 spectrophotometer and 2100 Bioanalyzer. Equal amounts of RNA from different tissues (mature leaf, flower, dwarf stem, root, and receptacle tissues, from six different stages) were combined for SMRT sequencing, and the total RNA from red fruit of two strawberry varieties (Xiaobai and Benihoppe) was used for Illumina sequencing.

### PacBio cDNA library preparation and SMRT sequencing

Synthesis of cDNA was performed using a SMARTer^TM^ PCR cDNA Synthesis Kit (Takara Clontech Biotech, Dalian, China). Size fractionation and selection (1–2 kb, 2–3 kb, and >3 kb) was performed using the BluePippin™ Size Selection System (Sage Science, Beverly, MA). The three libraries were generated using Pacific Biosciences DNA Template Prep Kit 2.0 following the standard protocol. The 1–2 and 2–3 kb libraries were derived from sequencing one SMRT cell sample, and the >3 kb library was from two SMRT cell samples on the Pacific Bioscience RS II platform.

### Illumina cDNA library construction and second-generation sequencing

The Illumina cDNA libraries were generated by a NEBNext^®^ Ultra™ RNA Library Prep Kit (NEB, Beverly, MA, USA). Qualified libraries were sequenced on the Illumina Hiseq 2500. In total, 58.76 Gb of 125-bp paired-end reads were generated.

### SMRT sequencing data processing and error correction

The SMRT-Analysis software package v2.3.0 was used for the analysis of SMRT sequencing data. Reads of inserts were extracted with the minimum filtering requirement of 0 or minimum accuracy of 75. Non-chimeric ROI sequences were divided into full-length ROIs (the presence of poly(A) tail signals, 5′ adaptors, and 3′ adapter sequences in the ROIs) or non-full-length ROIs. Furthermore, full-length ROIs were passed through isoform-level clustering (ICE) to obtain consensus isoforms, and full-length consensus sequences from ICE were polished using the Quiver software module. High-quality (HQ) isoform sequences with a post-correction accuracy greater than 99% were generated for further analysis. Low-quality (LQ) isoform sequences with a postcorrection accuracy lower than 99% were further corrected using Illumina reads as the reference with the software proovread v2.13.13^43^. After combining the HQ and LQ transcripts, redundant isoforms were then removed using CD-HIT 4.6.1 software^44^. The rest of the transcripts were searched against themselves by a BLASTN (v2.2.26) search to eliminate chimeric reads. We also performed a BLASTX (v2.2.26) (cutoff E-value ≤ 1e^−5^) search against the NR (NCBI nonredundant proteins) database to further remove redundant transcripts and transcripts that were not from plants, such as bacteria, fungi, and insects. Therefore, we generated a high-quality transcript dataset for cultivated strawberry (*Fragaria* x *ananassa*), which we call the “SMLR”.

### LncRNA identification from PacBio sequences

We built a combination of four pervasive coding potential assessment approaches, including coding potential calculator (CPC v1)^45^, coding-noncoding index (CNCI v2)^46^, coding potential assessment tool (CPAT v1.2)^47^, and the Pfam^48^ database 27.0 to identify long noncoding RNA (lncRNA) candidates from SMLR isoforms.

### Characterization of AS events

The SMLR isoforms were mapped to the *Fragaria vesca* Genome v4.0.a1 by GMAP (version 2017-11-15) with >85% alignment coverage and >90% alignment identity^49^. Five major types of AS events, including mutually exclusive exon, intron retention, skipped exon, alternative 5′ splice sites, and alternative 3′ splice sites, were identified by Astalavista v3.2 (http://sammeth.net/confluence/display/ASTA/Home) ^50^.

### Transcript annotation

Functional annotation of the isoforms in SMLR was conducted against a series of public databases using BLASTX (v2.2.26) (cutoff E-value ≤ 1e^−5^)^51^, including COG (Clusters of Orthologous Groups), KEGG (Kyoto Encyclopedia of Genes and Genomes), KOG (Clusters of Orthologous Groups of proteins), Pfam (a database of conserved protein families or domains), Swiss-prot (a manually annotated and reviewed protein sequence database), and NR (NCBI nonredundant proteins). The functional information of the best matched sequence was then assigned to the query isoforms in SMLR. Functional classification by GO (gene ontology) analysis of the isoforms in SMLR was performed using the program Blast2GO (v2.5). Through GO annotation, the isoforms were classified into the following terms of the three GO categories: (BP) biological process, (CC) cellular component, and (MF) molecular function.

### Transcriptome completeness analysis

A set of 1440 conserved Plantae orthologous proteins was used in BUSCO (Benchmarking Universal Single-Copy Orthologs) v3.0.2^52^ to assess the completeness of the SMLR, RefTrans^36^, and FANhybrid_r1.2.cds ^53^.

### Description of RNA-seq datasets from NCBI

The RNA-seq datasets were acquired from the Sequence Read Archive (SRA) at NCBI (http://www.ncbi.nlm.nih.gov/sra). The accession number was SRP111905. The RNA-seq datasets included four samples (each with two biological replicates) representing four successive developmental stages (green fruit, GF; white fruit, WF; turning stage, TS; and red fruit, RF) of strawberry (*Fragaria* x *ananassa*) fruit.

### Illumina data analysis

We first mapped the clean reads to the SMLR isoforms using Bowtie2 (v 2.2.4)^54^, and the unmapped reads were extracted. Transcript expression levels were quantified by FPKM. Differential expression analysis was performed using the DESeq R package^55^. The false discovery rate was controlled by adjusted *P-*values using Benjamini and Hochberg’s method^56^. Transcripts with FC ≥ 2 and FDR ≤ 0.01 were defined as differentially expressed. K-means clustering was conducted based on Pearson correlation of gene expression profiles ^57^.

### Identification of TFs, auxin and ABA pathways, and anthocyanin biosynthesis isoforms in SMLR

*F. vesca* TF protein sequences acted as the reference, which was downloaded from Plant TFDB 4.0^58^. The isoforms in SMLR were used as a query to search the *F. vesca* TF protein database by BLASTX (e-value ≤ e^−5^). Filter criteria (coverage >80%; identity >90%) were applied to detect TFs in SMLR, which were subsequently searched against the NR database to confirm whether they were TFs (highest bit score).

The *F. vesca* auxin pathway, ABA pathway, and anthocyanin biosynthesis genes were searched by a BLAST (v2.2.26) search against the *Fragaria vesca* gene models (*Fragaria vesca* Genome v2.0.a1) using *Arabidopsis* protein sequences as queries. The top hits were then compared against the NR database to confirm them as strawberry (*Fragaria vesca*) auxin pathway, ABA pathway, and anthocyanin biosynthesis genes by BLAST. After eliminating the isoforms with a premature stop from alternative splicing events, the SMLR isoforms mapped to those *F. vesca* genes were designated as strawberry (*Fragaria* x *ananassa*) auxin pathway, ABA pathway, and anthocyanin biosynthesis transcripts.

## Accession numbers

Raw reads of one combined SMRT library and one Illumina RNAseq library generated in this study are available from the BioProject at NCBI (https://www.ncbi.nlm.nih.gov/bioproject/) under the accession numbers PRJNA510223 and PRJNA510532, respectively.

## Supplementary information

Supplementary Information 1

Supplementary Information 2

Supplementary Information 3

Supplementary Information 4

Supplementary Information 5

Supplementary Information 6

Supplementary Information 7
