# Supplementary material for: The complexity of the Fragaria x ananassa (octoploid) transcriptome by single-molecule long-read sequencing
Source: Hortic Res. 2019 Apr 6;6:46. doi: 10.1038/s41438-019-0126-6 (PMC6441658; doi:10.1038/s41438-019-0126-6)
Supplement: Supplementary file 1 — Supplementary Information 1 [file 41438_2019_126_MOESM1_ESM.docx]

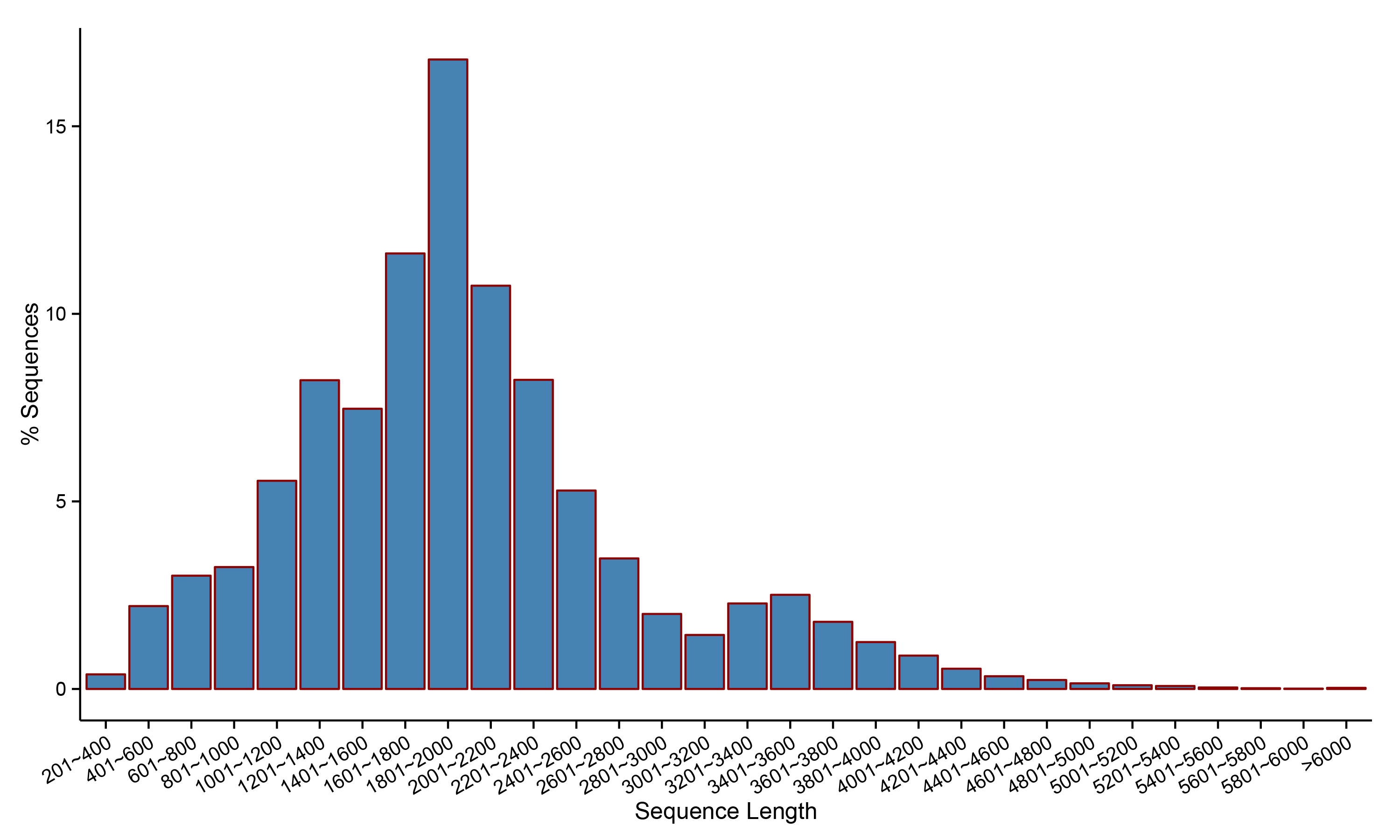


Figure S1. Length (bp) distribution of FLNC reads


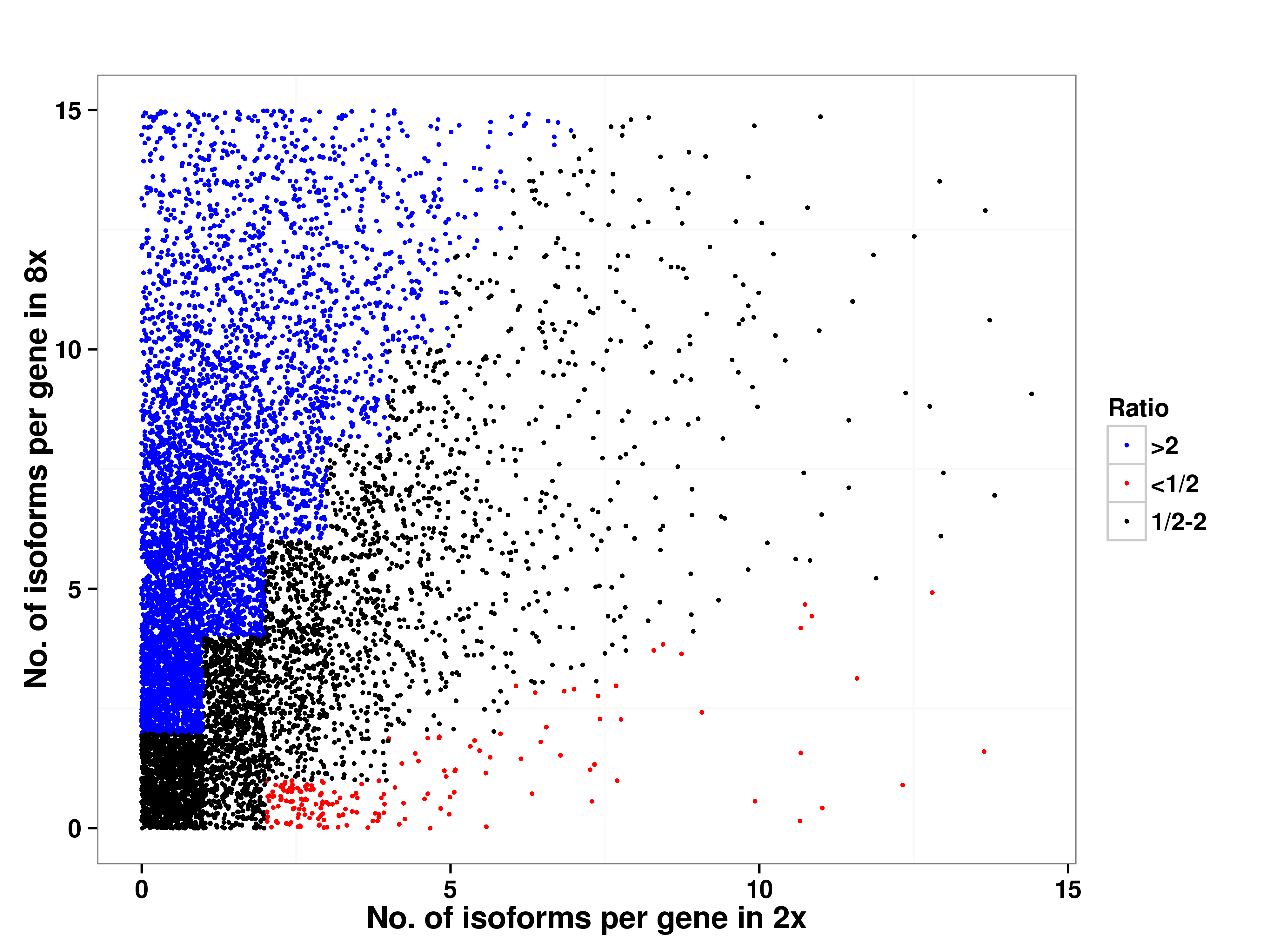


Figure S2. Scatterplot showing the number of isoforms per annotated gene in SMLR and *Fragaria vesca* PacBio Iso-Seq.


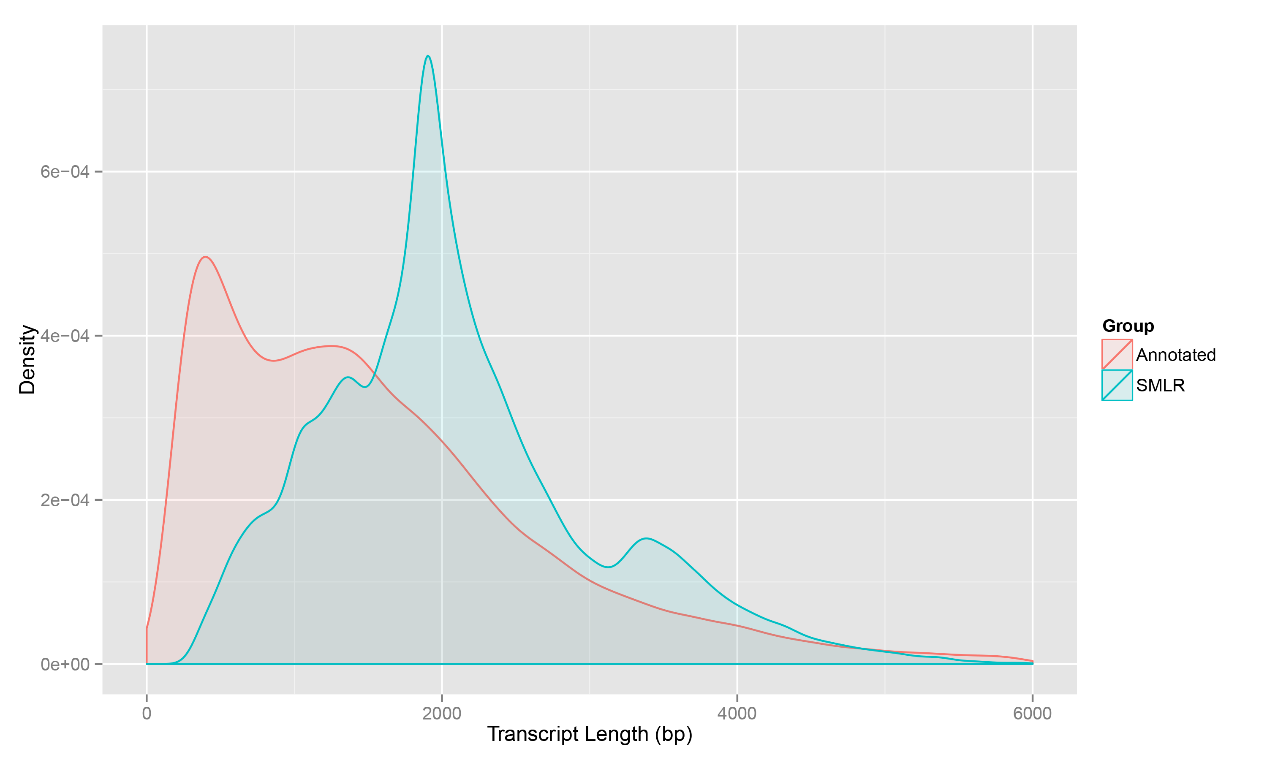


Figure S3. Density distribution of transcript length in SMLR and the corresponding annotated *Fragaria vesca* gene models.


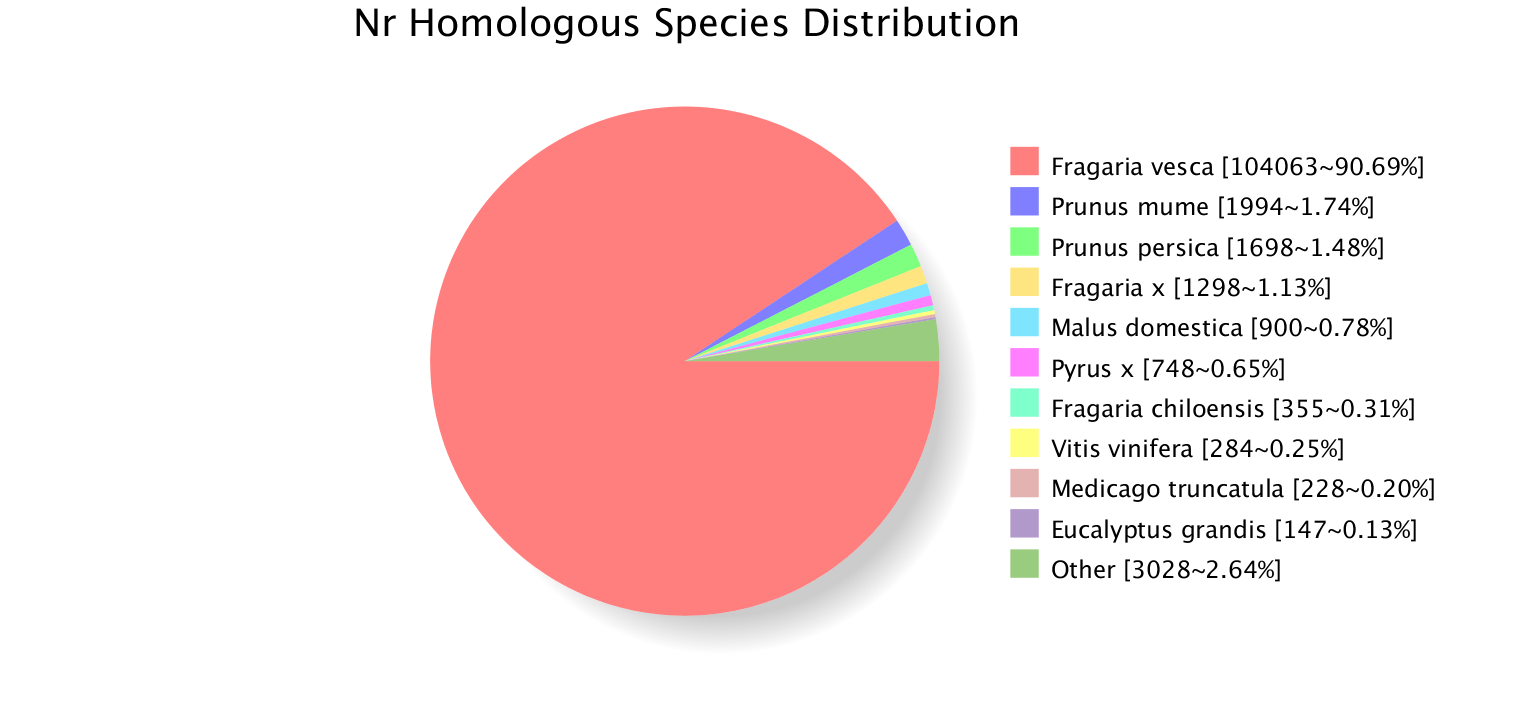


Figure S4. Top-Hit Species distribution of SMLR isoforms according to the result of BLASTx against NR database.


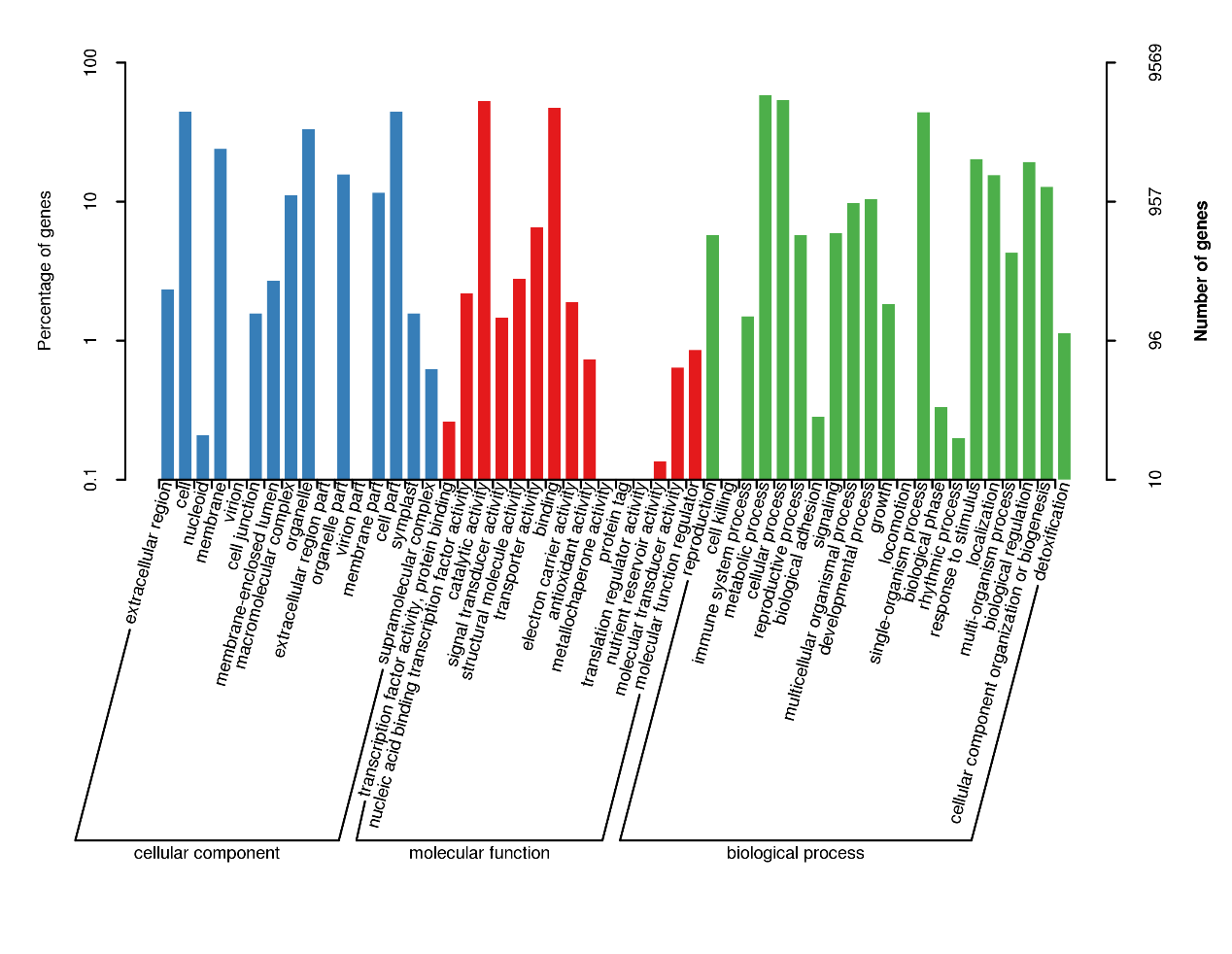


Figure S5. Functional classification of SMLR isoforms by using Gene Ontology (GO) terms.


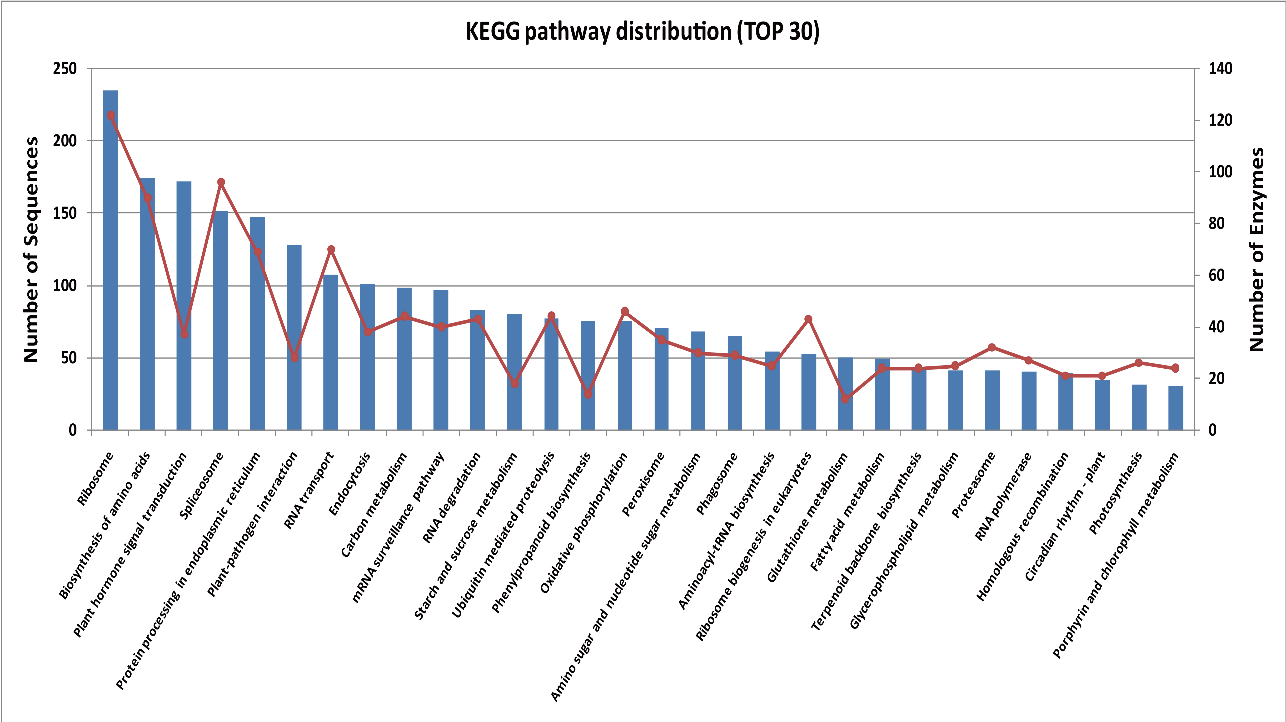


Figure S6. The KEGG pathway distribution of SMLR isoforms.


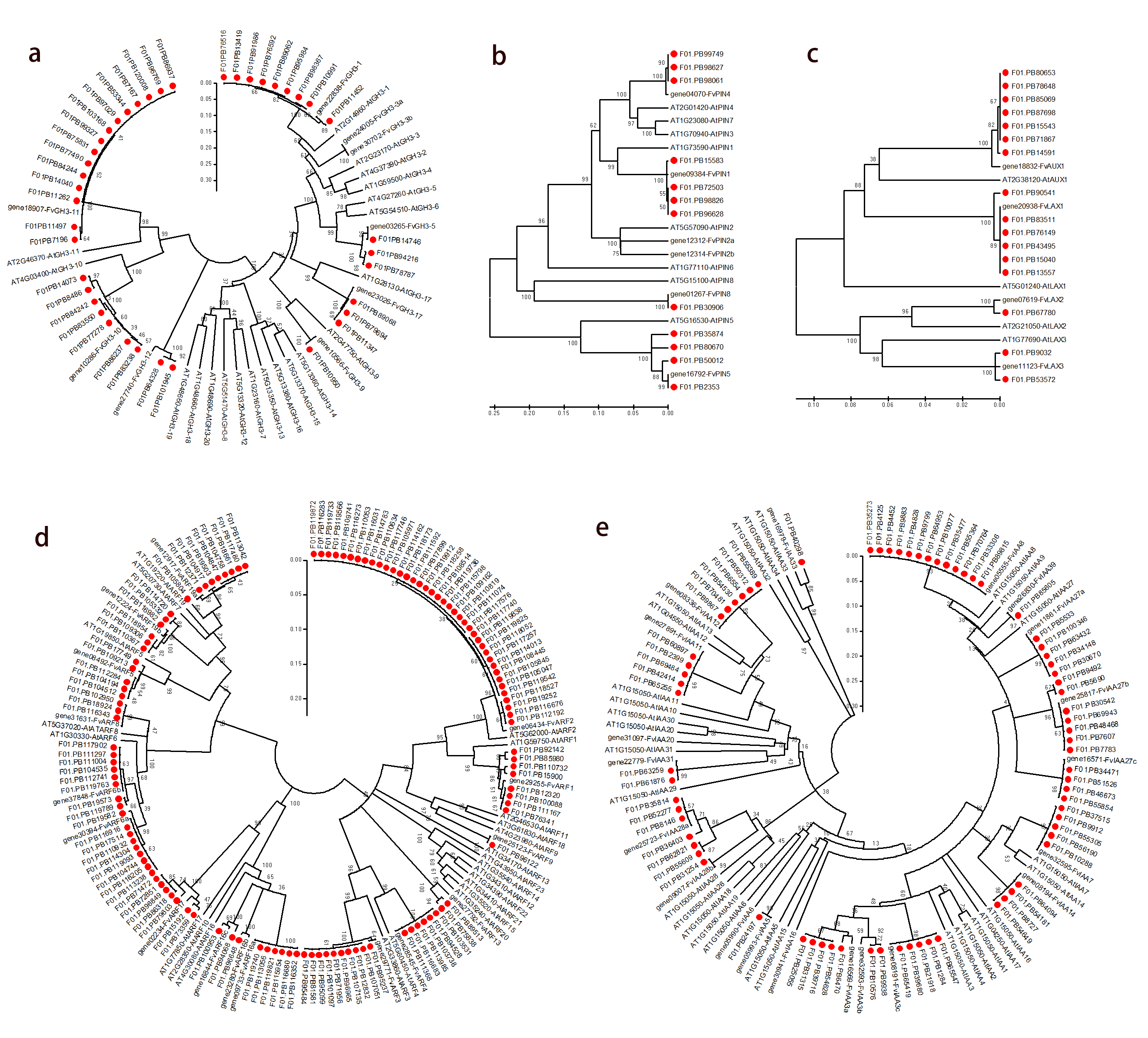


Figure S7. Phylogenetic trees of major auxin protein sequences.

The protein sequences of GH3, PIN, AUX/LAX, ARF, and AUX/IAA families in *Arabidopsis* and *F. vesca* are downloaded from GDR (https://www.rosaceae.org/) and TAIR (https://www.arabidopsis.org) databases. The candidate isoforms in SMLR were confirmed by BLAST against Non-redundant protein sequences (nr) in NCBI. Genes in *F. vesca* are based on previous publications (Kang et al., 2013). Phylogenetic trees were made by neighbor-joining with a bootstrap test with 1000 iterations in MEGA5.


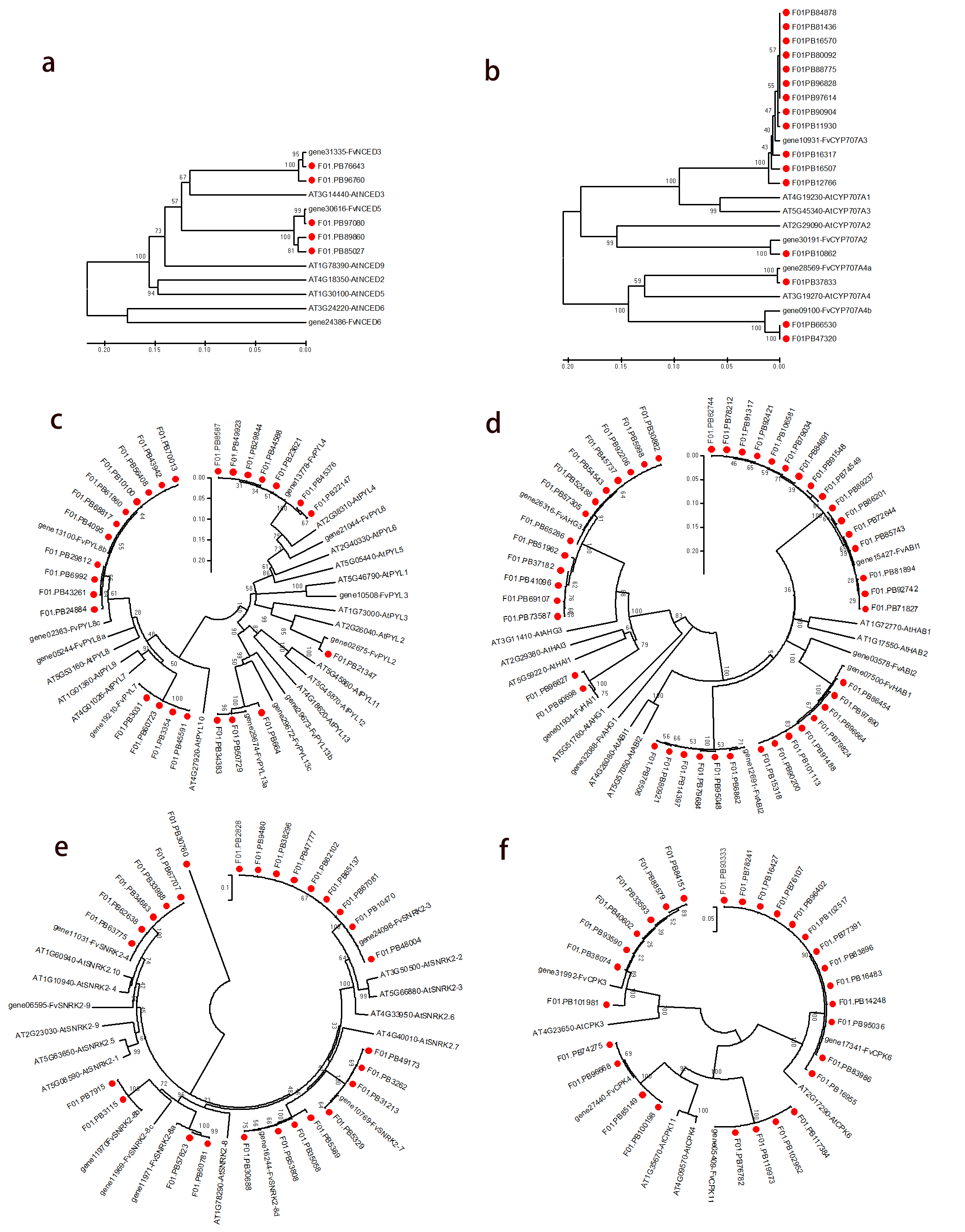


Figure S8. Phylogenetic trees of major ABA protein sequences.

The protein sequences of NCED, CYP707A, PYL, PP2C, SNRK2, and CPK families in *Arabidopsis* and *F. vesca* are downloaded from GDR (https://www.rosaceae.org/) and TAIR (https://www.arabidopsis.org) databases. The candidate isoforms in SMLR were confirmed by BLAST against Non-redundant protein sequences (nr) in NCBI. Genes in *F. vesca* are based on previous publications (Kang et al., 2013). Phylogenetic trees were made by neighbor-joining with a bootstrap test with 1000 iterations in MEGA5.


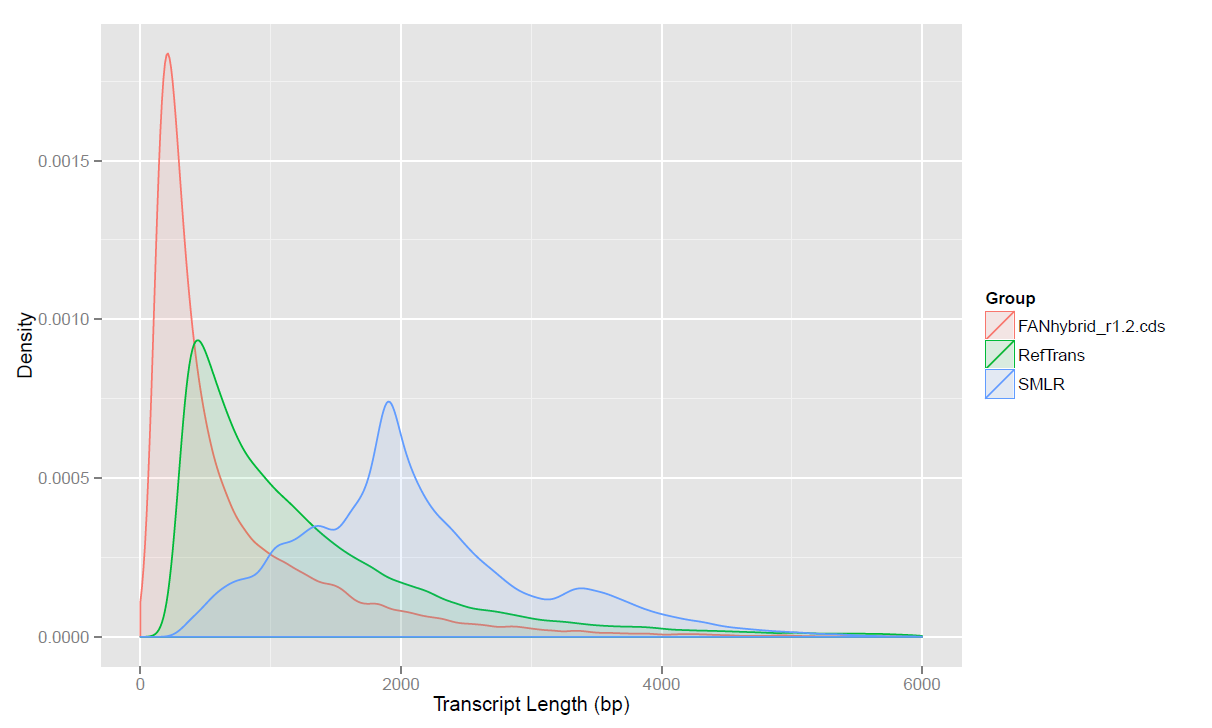


Figure S9. Density distribution of transcript length (< 6000bp) in FANhybrid_r1.2.cds, RefTrans and SMLR.


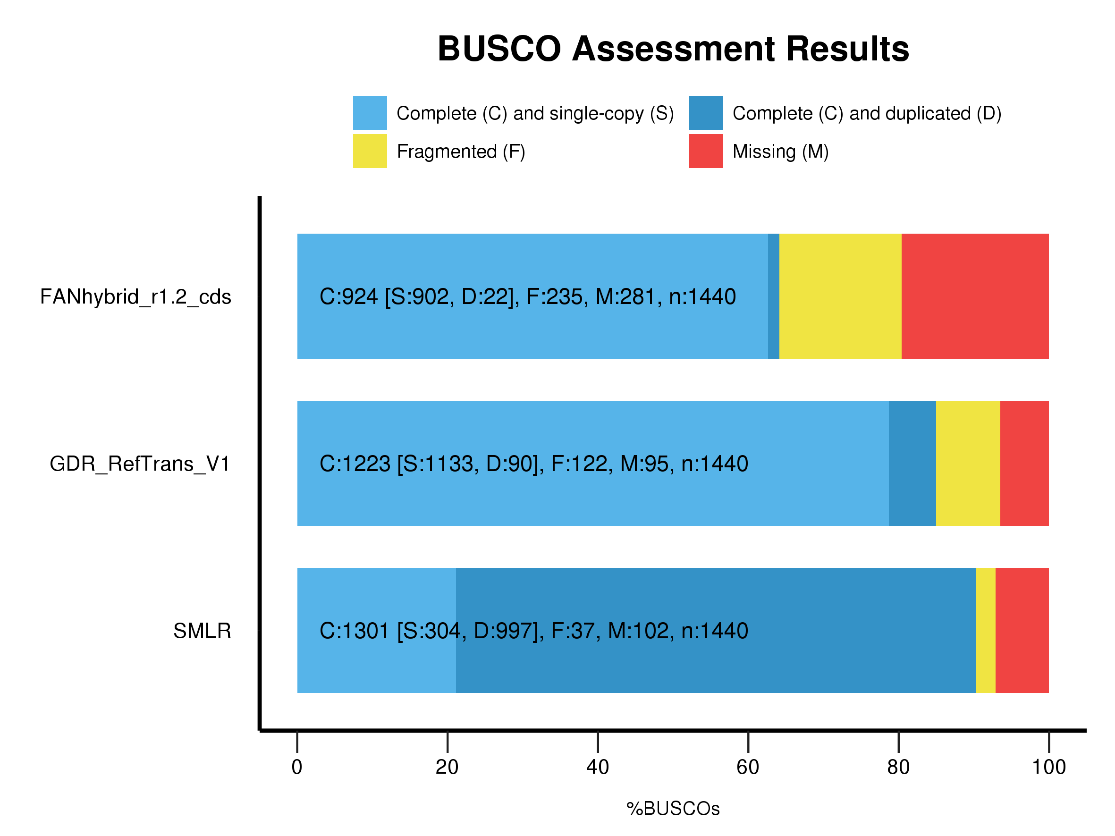


Figure S10. Transcriptome coverage analysis of FANhybrid_r1.2.cds, RefTrans and SMLR based upon BUSCO alignment.

Table S1. The statistical result of RNA-seq reads mapped to isoforms of SMLR by Bowtie2

| BMK-ID | Total Reads | Mapped Reads(%) | Uniq mapped Reads(%) | Multi mapped Reads(%) |
| --- | --- | --- | --- | --- |
| GF1 | 29,270,918 (100%) | 24,274,149 (82.93%) | 1,885,200 (7.77%) | 22,388,949 (92.23%) |
| GF2 | 30,290,933 (100%) | 25,068,814 (82.76%) | 2,028,120 (8.09%) | 23,040,694 (91.91%) |
| WF1 | 33,348,481 (100%) | 27,949,850 (83.81%) | 2,008,572 (7.19%) | 25,941,278 (92.81%) |
| WF2 | 30,891,550 (100%) | 25,751,436 (83.36%) | 1,950,386 (7.57%) | 23,801,050 (92.43%) |
| TS1 | 31,540,687 (100%) | 26,624,124 (84.41%) | 1,956,603 (7.35%) | 24,667,521 (92.65%) |
| TS2 | 30,672,534 (100%) | 25,826,571 (84.20%) | 1,759,525 (6.81%) | 24,067,046 (93.19%) |
| RF1 | 30,923,599 (100%) | 25,933,370 (83.86%) | 1,946,212 (7.50%) | 23,987,158 (92.50%) |
| RF2 | 29,789,512 (100%) | 24,909,940 (83.62%) | 1,825,257 (7.33%) | 23,084,683 (92.67%) |

Note: Mapped Reads(%): percentage of mapped reads to isoforms in SMLR; Uniq mapped Reads(%): percentage of mapped reads with only one mapped isoforms in SMLR; Multi mapped Reads(%): percentage of mapped reads with only two or more mapped isoforms in SMLR.
